# Ancient oncogenesis, infection and human evolution

**DOI:** 10.1111/eva.12497

**Published:** 2017-07-11

**Authors:** Riaan F. Rifkin, Marnie Potgieter, Jean‐Baptiste Ramond, Don A. Cowan

**Affiliations:** ^1^ Center for Microbial Ecology and Genomics (CMEG) Department of Genetics University of Pretoria Hatfield South Africa

**Keywords:** ancient DNA, *Australopithecus*, *Homo*, oncogenesis, pathogens, Pleistocene, sub‐Saharan Africa

## Abstract

The recent discovery that malignant neoplastic lesions date back nearly 2 million years ago not only highlights the antiquity of cancer in the human lineage, but also provides remarkable insight into ancestral hominin disease pathology. Using these Early Pleistocene examples as a point of departure, we emphasize the prominent role of viral and bacterial pathogens in oncogenesis and evaluate the impact of pathogens on human evolutionary processes in Africa. In the Shakespearean vernacular “what's past is prologue,” we highlight the significance of novel information derived from ancient pathogenic DNA. In particular, and given the temporal depth of human occupation in sub‐Saharan Africa, it is emphasized that the region is ideally positioned to play a strategic role in the discovery of ancient pathogenic drivers of not only human mortality, but also human evolution. Ancient African pathogen genome data can provide novel revelations concerning human‐pathogen coevolutionary processes, and such knowledge is essential for forecasting the ways in which emerging zoonotic and increasingly transmissible diseases might influence human demography and longevity in the future.

## 1 INTRODUCTION

Advances in ancient DNA (aDNA) research and the detection of prehistoric bacterial, viral and fungal pathogens has rapidly advanced our understanding of the antiquity of human‐pathogen interactions (Bos et al., 2011, 2015; Devault et al., 2014; Harkins & Stone, 2015; Louvel, Der Sarkissian, Hanghøj, & Orlando, 2016; Pimenoff, de Oliveira, & Bravo, 2016; Rasmussen et al., 2015; Schuenemann et al., 2013). The analyses of phylogenetic relationships of extant pathogens furthermore suggest that many diseases have been coevolving with humans for millennia. In addition to the classic parasite–host coevolutionary contest typified by the link between the malaria‐causing *Plasmodium falciparum* parasite and the origin of HbS sickle‐cell disease at *c*. 100,000 years ago (ka) (Kwiatkowski, 2005), the antiquity of genetic disease prevention mechanisms, such as the origin of immune‐regulating Sia‐recognizing Ig‐like lectin (SIGLEC) genes before 70 ka (Wang et al., 2012), confirms that pathogens played an essential role in human evolution in Africa. Additional examples confirming long‐standing exposure to pathogens include the incidence of *Helicobacter pylori* amongst human populations for >60,000 years (Moodley et al., 2012), evidence for the coevolution of *Mycobacterium tuberculosis* and humans from 70 ka (Comas et al., 2013) and recent indications that human papillomavirus (HPV) coevolved with ancestral Africans from at least *c*. 500 ka (Pimenoff et al., 2016). As infectious agents are recognized as selective agents for human polymorphisms, strong selection by pathogens (i.e., interactions of infectious agents with the innate immune system) is expected to be implicated in the evolution of our species (Wang et al., 2012). Pathogens (e.g., *H. pylori*), and also human parasites (i.e., human lice) have furthermore been used to track human population movements and have provided valuable evidence regarding human migrations within and out of Africa (Comas et al., 2013; Moodley et al., 2012; Reed, Smith, Hammond, Rogers, & Clayton, 2004) and into the New World (Raoult et al., 2008). But exactly which disease vectors and pathogens were brought from Africa to the rest of the world following the departure of behaviourally modern *Homo sapiens* (BMHs) from the continent after *c*. 100 ka (Reyes‐Centeno, Hubbe, Hanihara, Stringer, & Harvati, 2015), remains unclear. While small, itinerant prehistoric foraging groups could not sustain a broad range of epidemic infectious agents (e.g., measles and influenza), it is nevertheless from this pre‐65 ka sub‐Saharan African “Pleistocene disease baseline” that most modern diseases derive. Indeed, current evidence suggests that at least 20 modern human diseases have certain to probable African origin, including hepatitis B, measles, HIV, Kaposi's sarcoma‐associated herpesvirus (KSHV), HPV, cholera, dengue fever, sleeping sickness, *P. falciparum* and *Plasmodium vivax* malarias, leishmaniasis, plague and smallpox (Harkins & Stone, 2015; Houldcroft & Underdown, 2016; Trueba & Dunthorn, 2012; Wolfe, Dunavan, & Diamond, 2007). Many of these had a profound influence on human evolutionary history, and most of the above are still implicated in the deaths of millions of people annually.

Although recent assessments of prehistoric pathogen prevalence are providing increasingly informed perspectives on the taxonomic variety and geographic origins of diseases (Harkins & Stone, 2015), these derive largely from European and Near‐Eastern Mediaeval and Holocene contexts. The analysis of ancient pathogenic DNA (apDNA) from prehistoric African contexts is lacking. As the region forms the focus of early modern human evolutionary research, one would expect the subcontinent to play a prominent role in aDNA research. African human and pathogenic aDNA is, after all, crucial to the reconstruction of the evolutionary history of anatomically modern humans (Slatkin & Racimo, 2016). While this has not yet materialized, and although the field is dominated by a few well‐funded and highly specialized European laboratories, the role of sub‐Saharan Africa in both a prehistoric and current global epidemiological context cannot be underestimated. Specifically, and given the temporal depth of human occupation in southern Africa, and its vast ecological and geographic diversity, the region is ideally positioned to play a strategic role in the exploration and discovery of past pathogenic drivers of human mortality. Accordingly, we present an up‐to‐date overview of research concerning prehistoric oncogenesis, highlighting the importance of ancient human evolutionary perspectives using aDNA to better understand modern oncogenic pathogen diversity and dynamics. We also explore the widely held misconception that pathogen‐driven oncogenesis was rare or nonexistent in human prehistory, and emphasize the essential role of sub‐Saharan African archaeological contexts in elucidating the evolutionary impact of oncogenic and other bacterial and viral pathogens on the evolution of our species in Africa.

## 2 ANCIENT HUMAN HEALTH AND ONCOGENESIS

Epidemiologic transition models generally associate the emergence of most human diseases with changing living conditions resulting from agricultural innovations and higher population densities that occurred during the Neolithic Period, *c*. 12 ka (Omran, 1971). Consequently, the search for the origins of diseases has focussed largely on domestic animals and environments outside Africa. Many of these tropical infections are, however, likely to have played a significant role in the human evolutionary process for much lengthier periods of time (Barrett, Kuzawa, McDade, & Armelagos, 1998). It is conceivable that the original state of human disease exposure is characterized by the prehistoric sub‐Saharan African populations who inhabited the region over the past 150,000 years. The potential impact of disease on prehistoric humans is illustrated by the fact that ~60% of contemporary hunter‐gatherers succumb to disease before reaching reproductive age (*c*. 15 years) (Gurven & Kaplan, 2007). But, as indicated by the seminal review by Wolfe et al. (2007), and more recently those by Harkins and Stone (2015), Houldcroft and Underdown (2016) and Trueba and Dunthorn (2012), there are substantial discontinuities in our understanding of the origins of diseases and their influence on human evolution in Africa.

The current global disease burden is dominated by both ancestral (Houldcroft & Underdown, 2016; Wolfe et al., 2007) and novel emerging or re‐emerging infectious diseases (Langwig et al., 2015; Plummer et al., 2016). Of the ~2,100 species of pathogens that affect humans (Wardeh, Risley, McIntyre, Setzkorn, & Baylis, 2015), 65% are zoonotic (Lloyd‐Smith et al., 2009) and 177 (8.4%) cause emerging infectious diseases (Dutour, 2013). Of all the illnesses afflicting modern human society, cancer arguably represents one of the most enigmatical ailments (Boyle & Levin, 2008; Hanahan & Weinberg, 2000). In 2015, noncommunicable neoplasms (new and abnormal tissue growths characteristic of cancer) were a leading cause of the global disease burden (Kassebaum et al., 2016). Disability‐adjusted life‐years indices indicate that neoplasms were implicated in ~215 million years of life lost due to either death or disability. Neoplastic diseases were surpassed in impact only by cardiovascular and other infectious diseases. But are neoplastic diseases restricted to postindustrial human society, or can we trace the origins of malignant cancerous tumours further back in time, perhaps even into prehistory?

Citing the rarity of hominin fossil evidence for oncogenic tumours, David and Zimmerman (2010) recently concluded that cancer is a contemporary human phenomenon that is caused by the stresses of our modern lifestyle. Changes in diet and anthropogenic environmental modification are proposed to have subjected humans to toxins that contribute to cancers. Consequently, a widely held and highly erroneous perception is that the increase in and risk of contracting cancer is driven almost exclusively by anthropogenic, environmental and, to a lesser extent, inheritable (genetic) factors. But referring to a lack of evidence for the occurrence of cancer in the hominin archaeological record as indicative of the paucity of malignancies in antiquity is erroneous. Nearly all palaeopathological examples of cancer only dates to the past 500 years of human history, and evidence for cancer before the modern era is indeed rare (Binder, Roberts, Spencer, Antoine, & Cartwright, 2014). Early confirmation of neoplastic disease is however indicated by a lesion on an archaic *Homo* mandible from Kanam, Kenya (Phelan et al., 2007), and a fibrous dysplasia on a Neanderthal rib dated to 120 ka from the site of Krapina, Croatia (Monge et al., 2013). The recent discovery of neoplastic tumours in members of *Australopithecus* and early *Homo* (Odes et al., 2016; Randolph‐Quinney et al., 2016) dated to 1.98 and *c*. 1.7 million years ago, respectively, provides additional insight into the antiquity of human cancers. These two remarkable Early Pleistocene South African finds also necessitate a revision of current perceptions regarding the causative factors implicated in oncogenesis.

**Figure 1:**
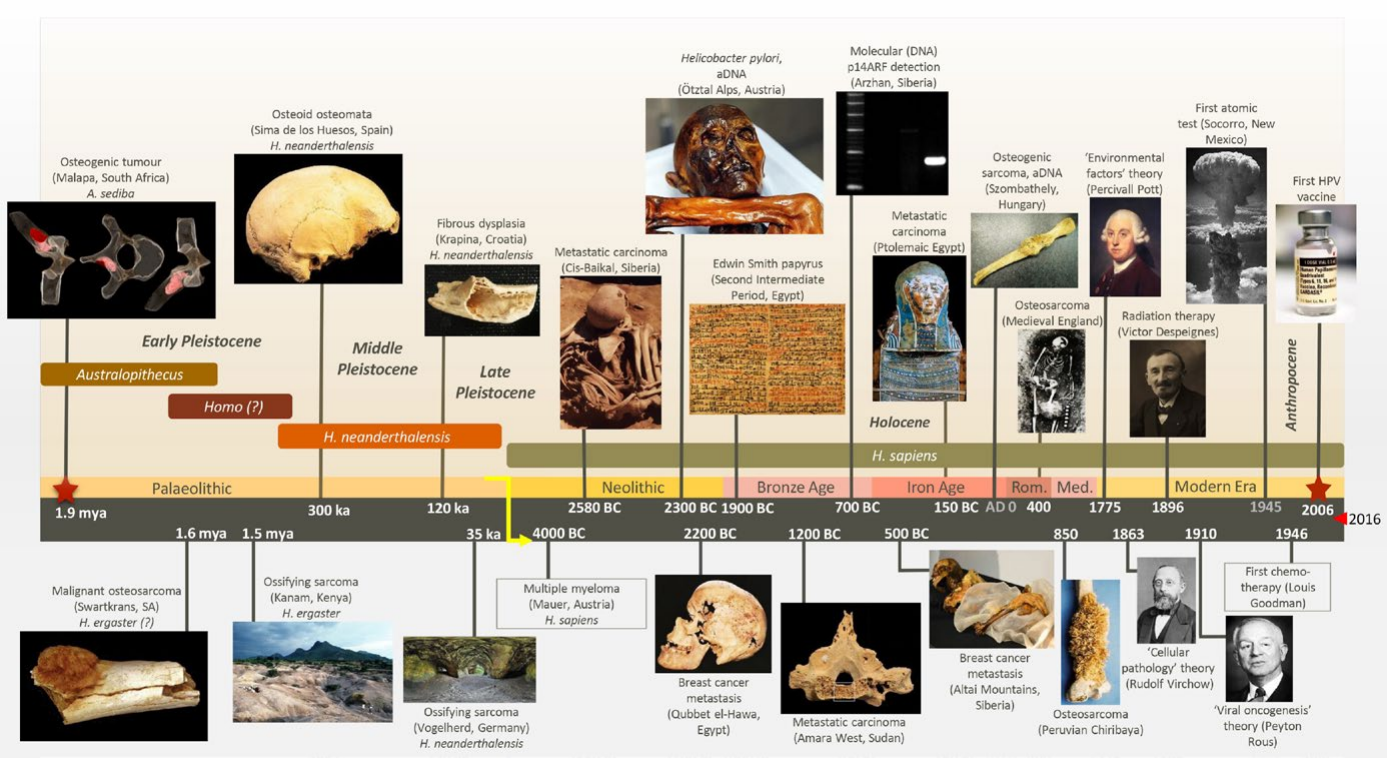
Chronological incidence of prehistoric oncogenic tumours and important milestones concerning cancer aetiology and treatment (Binder et al., 2014; Bona et al., 2014; Monge et al., 2013; Odes et al., 2016; Phelan et al., 2007; Randolph‐Quinney et al., 2016) (‘Rom.’ and ‘Med.’ referes to Roman and Medieval Periods, respectively).

Admittedly, individuals who succumb to death shortly after oncogenesis will not display skeletal indications of either benign or malignant cancer tumours (i.e., osteosarcoma, chondrosarcoma and multiple myeloma), while those that did survive long after the formation of tumours might, in some instances, have developed skeletal lesions (Brothwell, 2016). In addition, extraskeletal tumours leave absolutely no signs of their existence on human osseous remains. The rarity of prehistoric human remains further obscures our perception of cancer incidence in ancient times. An online evaluation of human fossil evidence for the past 100,000 years indicates that there are only about 50 *H. sapiens* (~28) and *Homo neanderthalensis* (~23) skeletal examples available for palaeopathological analysis (http://www.humanoriginsdatabase.org/). Most of these specimens comprise fragmented examples representative of incomplete skeletons, and only two display skeletal morphologies that relate to malignant tumorous development (Figure 1). Thus, and on account of this “osteological paradox” (Wood et al., 1992), disease incidence is often unnoticed or misconstrued, which leads to unverified statements that some diseases were either rare or nonexistent in prehistory.

## 3 INTRINSIC AND EXTRINSIC FACTORS IMPLICATED IN ONCOGENESIS

The relative contribution of intrinsic (inheritable genetic) and extrinsic (environmental) risk factors in cancer development has been the subject of extensive scientific discussion (Lin et al., 2015; Luzzatto & Pandolfi, 2015; Ngeow & Eng, 2016; Pimenoff et al., 2016; Plummer et al., 2016; Tomasetti & Vogelstein, 2015; Wu, Lu, Zhou, Chen, & Xu, 2016; Wu, Powers, Zhu, & Hannun, 2016). Carcinogenesis or oncogenesis entails the process whereby normal cells are transformed into cancer cells. The progression is characterized by changes at the cellular, genetic and epigenetic levels and abnormal cell division which, in some cancers, can result in the formation of a malignant tumorous mass. Cancer cells typically acquire the ability to reproduce uncontrollably, thus resulting in the development of tumours. The underlying causative factors implicated in normal cell alterations are however highly variable, and many types of cancers arise from chronic wounds and at sites of infection and inflammation (Coussens & Werb, 2002).

Tomasetti and Vogelstein (2015) recently suggested that the risk of developing cancer is strongly correlated with the total number of divisions of stem cells in specific organs or tissues. Random mutations arising during DNA replication in noncancerous stem cells are cited as a primary cause of oncogenesis. Accordingly, patients with familial adenomatous polyposis syndrome are estimated to be ~30 times more likely to develop colorectal cancer than duodenal cancer, primarily because there are ~150 times as many stem cell divisions in the colon as in the duodenum. These and other mutational errors therefore arise by chance during stem cell division and is said to explain more cancers than do hereditary or environmental factors. Peculiarly, the underlying mechanism is ascribed to “bad luck” as imposed by the random stochastic mutation events that occur during DNA replication. Is oncogenesis simply down to “bad luck,” or are there other oncogenic mechanisms at work here?

Rudolf Virchow (1821–1902) first proposed the irritation hypothesis of carcinogenesis, positing that cancer development entailed the alteration of normal human cells. Following his observation of the inflammatory reaction in *Schistosoma*‐related bladder cancers, he suggested that chronic irritation triggered the development of malignant (cancer) cells (Balkwill & Mantovani, 2001). Accordingly, the inflammatory process is characterized by damage caused by the host immune response to the infection, rather than by the infecting organism itself. More than a century after Virchow's findings, the “chronic irritation hypothesis” remains a widely supported mechanism for carcinogenesis by infectious agents. Genetic variations also influence the likelihood of developing a particular type of cancer. Inheritable mutations or cancer‐predisposing genes that increase the risk of cancer may be passed on from parent to child. While these genetic changes may well contribute to the development of cancer, they do not directly cause it. An estimated 5%–10% of all cancers are heritable, meaning that a single gene mutation contributes to the development of cancer (Ngeow & Eng, 2016). For breast cancer, a leading cause of cancer‐related death in women, the most important genes implicated are BRCA1 and BRCA2. These mutations are however only responsible for 10%–20% of cancer cases in patients with early‐onset or a family history of breast cancer (Lin et al., 2015). Mutations in the TP53 gene are one of the most frequent genetic alterations in human cancers (Olivier, Hollstein, & Hainaut, 2010). TP53 is a tumour suppressor and occurs at rates ranging from 38% to 50% in oesophageal, ovarian, colorectal, lung and larynx cancers to ~5% in primary leukaemia, sarcoma, testicular cancer, malignant melanoma and cervical cancer. Long‐term exposure to environmental carcinogens, including tobacco smoke, UVR exposure, vinyl chloride and herbal compounds derived from some species of plants (e.g., *Aristolochia*) comprise four well‐documented examples of associations between an aetiologic agent and the TP53 tumour mutation.

The fact that cancer incidence varies significantly amongst populations, organs and tissue types renders the prognostic accuracy of most (but not all) risk prediction models inadequate (Wang et al., 2015). Most of these cannot completely elucidate tumour occurrence by known potential determinants, such as environmental exposure, pathogens or inherited genes (Arnal et al., 2015). Luzzatto and Pandolfi (2015) recently highlighted the combined influence of stem cell turnover rates, stochastic mutation and exposure to known environmental mutagens in the development of cancer. Oncogenesis is dependent on various interacting and often anonymous variables, including age, sex, ethnic origin, geographic location, inheritance of susceptibility genes, obesity status, exposure to carcinogens, lifestyle idiosyncrasies and hormonal status, to pinpoint accurately, signifying inconstant and multifaceted mechanisms for cancer aetiologies.

In summary, current evidence indicates that intrinsic risk factors contribute only modestly (<10%–30%) to the lifetime risk of cancer development. On the contrary, the majority of cancers (70%–90%) can be ascribed to extrinsic environmental factors. Examples of environmentally induced cancers comprise colorectal cancer, with an estimated 75% of risk attributable to diet, malignant melanoma with 65%–86% of risk ascribed to excessive exposure to the sun and oesophageal cancers, in which case 75% are initiated by tobacco and alcohol abuse (Wu, Lu, et al., 2016; Wu, Powers, et al., 2016). This, along with several up‐to‐date reports (Lin et al., 2015; Ngeow & Eng, 2016; Plummer et al., 2016; Wu, Powers, et al., 2016), provides direct evidence that environmental factors can, and frequently do, play an essential role in cancer incidence. But what exactly is implied by “extrinsic environmental” factors?

In addition to >110 environmental substances known to be highly carcinogenic to humans, the International Agency for Research on Cancer (http://www.iarc.fr/) classifies ~370 chemical compounds and microorganisms as “probably carcinogenic” to humans. Although the influence of infectious organisms on carcinogenesis requires considerable exploration (Jacqueline et al., 2017), ~45 species of bacteria (including *H. pylori*,* Chlamydia trachomatis*,* Salmonella typhi* and *Streptococcus bovis*), viruses (e.g., hepatitis B, C and D virus, Epstein–Barr virus, Kaposi's sarcoma herpesvirus and HPV) and parasitic microorganisms (e.g., *P. falciparum*,* Opisthorchis viverrini* and *Schistosoma haematobium*) are currently listed as recognized causal factors in oncogenesis. Several oncogenic viruses (Epstein–Barr virus, HPV, human T‐lymphotropic virus type 1 and KSHV) are responsible for pathogen‐specific cancers (Table 1).

**Table 1:** Examples of generally accepted and probable (indicated by asterisks) viral, bacterial and parasitic infectious microorganisms implicated in human oncogenesis (Na, not available; Cummins & Tangney, 2013; de Martel et al., 2012; Ewald & Swain Ewald, 2015; Holland et al., 2016; Jacqueline et al., 2017; McIntyre, 2005; Okuku et al., 2013; Plummer et al., 2016; Vandeven & Nghiem, 2014; zur Hausen, 2009)

Infectious agent	Taxonomic affiliation	Implicated in infection (%)	Oncogenesis expressed, tropism
*Bacteria*			
*Helicobacter pylori*	Helicobacteraceae	90	Gastric cancer, mucosa‐associated lymphoid tumours
*Chlamydia trachomatis*	Chlamydiaceae	Na	Cervical cancer
Chlamydophila pneumoniae*	Chlamydiaceae	Na	Pulmonary mucosa‐associated Lymphoid tissue (MALT) lymphoma
*Mycoplasma* sp.	Mycoplasmataceae	Na	Lung and ovarian cancer
*Salmonella typhi*	Enterobacteriaceae	Na	Gallbladder cancer
Streptococcus gallolyticus*	Streptococcaceae	Na	Colon and colorectal cancer
Streptococcus bovis*	Streptococcaceae	Na	Colon and colorectal cancer
*Viruses*			
Epstein–Barr virus (EBV4)	Gamma herpesvirus	100	Nasopharyngeal and gastric cancers, Hodgkin's lymphoma, Burkitt's lymphoma
Human papillomavirus (HPV)	Alpha papillomavirus	100	Cervical, penile, oropharyngeal and rectal cancers
Human T‐lymphotropic virus type 1 (HTLV‐1)	Deltaretrovirus	100	Adult T‐cell leukaemia, lymphoma
Merkel cell polyomavirus (MCPyV)	Polyomavirus	80	Merkel cell cancer
Hepatitis B virus (HBV)	Hepadnavirus	60	Liver (hepatocellular) cancer
Hepatitis C virus (HCV)	Flavivirus	30	Liver (hepatocellular) cancer
Kaposi's sarcoma‐associated herpesvirus (KSHV/HHV‐8)	Gamma herpesvirus	100	Kaposi's sarcoma
Human mammary tumour virus (HMTV)*	Retroviridae	40	Breast cancer
*Parasites*			
*Clonorchis sinensis*	Opisthorchiidae	Na	Liver cancer (cholangiocarcinoma)
*Schistosoma haematobium*	Schistosomatidae	40	Bladder cancer
*Opisthorchis viverrini*	Schistosomatidae	Na	Gallbladder and bladder cancer
*Plasmodium falciparum*	Plasmodiidae	Na	Pancreatic cancer, leukaemia

**Figure 2:**
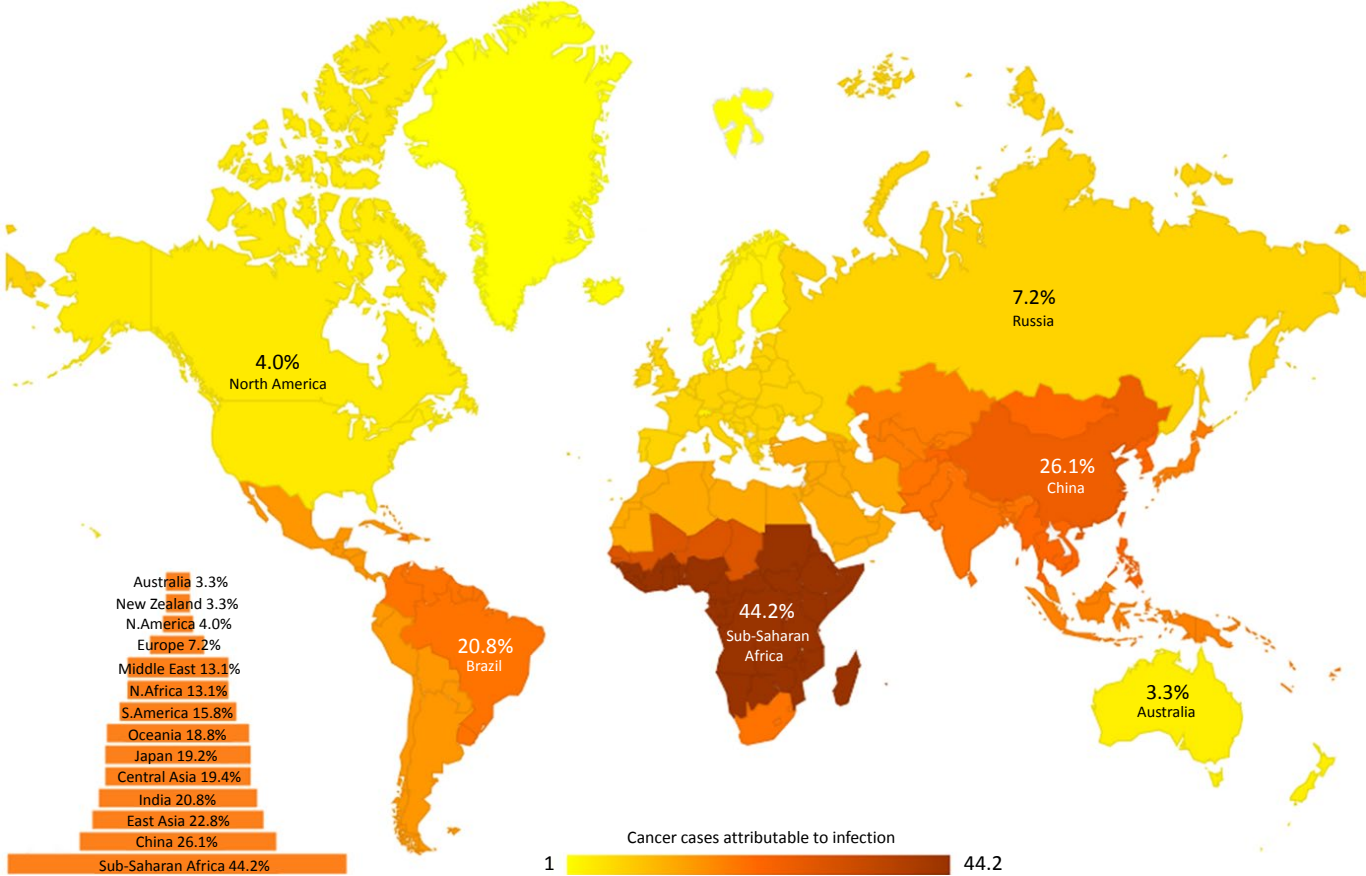
The global incidence of cancer attributable to pathogenic infection indicating the sizable (and approximate) proportion of infection‐related cases recorded in sub‐Saharan Africa (Cummins & Tangney, 2013; de Martel et al., 2012; Ewald & Swain Ewald, 2015; McIntyre, 2005; Okuku et al., 2013; Plummer et al., 2016; Vandeven & Nghiem, 2014; zur Hausen, 2009; http://canceratlas.cancer.org/risk-factors/infection/).

It is evident that the concept of cancer aetiology is incomplete without acknowledging the fundamental role of infection by pathogenic microorganisms (zur Hausen, 2008; de Martel et al., 2012). Globally, viral, bacterial and parasitic pathogens are implicated as causative factors in ~20% of cancer cases. But in sub‐Saharan Africa, 32.7% (de Martel et al., 2012; Plummer et al., 2016) to 40% (zur Hausen, 2009; McIntyre, 2005) of cancers are thought to be acquired through infections. Okuku et al. (2013) even suggest that as much as ~60% of cancer cases in sub‐Saharan Africa can be attributed directly to human immunodeficiency virus (HIV) infection. HIV is clearly an important infectious cofactor, despite the absence of a palpable aetiologic role. Because population‐based cancer registries cover only 11% of the sub‐Saharan African population, this alarming figure is probably an underestimate. In fact, when pooled, these figures amount to a startling 44.2% probability that viral, bacterial and parasitic pathogens are implicated directly in oncogenesis in sub‐Saharan Africa (Figure 2). And as infectious organisms not currently regarded as oncogenic may play a significant role in carcinogenesis (Jacqueline et al., 2017), it must be envisaged that the incidence of cancer in prehistory is also greatly underestimated.

## 4 PREHISTORIC HUMAN INTERACTION AND VIRAL ONCOGENESIS

If the general assumption that human cancers are caused primarily by lifestyle and environmental factors is accepted, how does one explain the incidence of cancer in preindustrialized societies? While increasing exposure to anthropogenic chemical carcinogens and dietary changes certainly does influence cancer aetiology, extrinsic environmental factors, in particular viral, bacterial and parasitic oncogenic pathogens, appears to play a primary role in cancer development. That oncogenesis has been in existence in the hominin lineage for at least 2 million years (Odes et al., 2016; Randolph‐Quinney et al., 2016) advocates adjustments in current onco‐pathogenic hypotheses. Cancer is a pathology of most multicellular organisms that appeared during the transition to metazoan life (multicellular, eukaryotic organisms) some 1 billion years ago. It is observed in just about the entire animal kingdom, from cnidarians to whales (Vittecoq et al., 2015).

More than 30 bacterial, viral and parasitic pathogens are implicated directly in oncogenesis, many of which are transmitted via sexual intercourse (Kassebaum et al., 2016; Plummer et al., 2016). Notable sexually transmitted infections (STIs) include chlamydia (*C. trachomatis*), gonorrhoea (*Neisseria gonorrhoeae*), syphilis (*Treponema pallidum*), HIV and trichomoniasis (*Trichomonas vaginalis*). The human sexual transmission pathway have also been implicated in the dissemination of re‐emerging pathogens such as Ebola virus disease (Deen et al., 2015) and Zika virus (Musso et al., 2015). HPV and KSHV (HHV‐8) are also transmitted via sexual contact. As indicated by current research (Kuhlwilm et al., 2016; Mondal et al., 2016; Sawyer, Krause, Guschanski, Savolainen, & Pääbo, 2012), there was certainly no lack of sexual encounters in prehistory. While witty epigrams coined by the popular media trivializes one of the most important events in the recent history of our species, the consequences of these chance encounters for modern human health cannot be underestimated.

Interspecies sexual interaction is not uncommon, and the recent genome sequencing of chimpanzees (*Pan troglodytes*) and bonobos (*Pan paniscus*) strengthens the idea that cross‐species mating also played an important role in the evolution of the great apes (de Manuel et al., 2016). For the genus *Homo*, it has been determined that Central African BiAka and Mbuti Pygmies, and southern and eastern African San, Hadza and Sandawe hunter‐gatherers contain a proportion of genetic material (~2%–5%) that introgressed from an archaic population *c*. 35 ka (Hammer, Woerner, Mendez, Watkins, & Wall, 2011; Hsieh et al., 2016; Lachance et al., 2012). Admittedly, unambiguous evidence of introgression in Africa is difficult to obtain as archaic African reference sequences do not yet exist. Comparative reference sequences are, however, available for Near‐Eastern and Eurasian populations. The admixture events involving *H. sapiens* and Neanderthals (*H. neanderthalensis*), Denisovans (*H. sapiens* ssp. “Denisova”) and an as yet unidentified archaic group (Kuhlwilm et al., 2016; Sawyer et al., 2012) had no bearing on sub‐Saharan African population genetics. As these events occurred in the Near East, Asia and Europe, sub‐Saharan Africans do not harbour any Neanderthal or Denisovan ancestry (Llorente et al., 2016; Sankararaman et al., 2014). Conversely, modern Eurasians inherited 1.5%–4% of their genomes from Neanderthals (Simonti et al., 2016) and ~5% from Denisovans (Sankararaman, Mallick, Patterson, & Reich, 2016). The behaviourally‐modern human (BMH) evolutionary history likely involved multiple regional admixture events with different, and some still unknown, archaic populations both in Africa and outside Africa (Hsieh et al., 2016; Mondal et al., 2016).

Although hybridization between diverging lineages (and closely related species) is not uncommon (Payseur & Rieseberg, 2016), Neanderthals and newly arriving BMHs were not biologically fully compatible. Whereas some Neanderthal alleles (variant forms of any given gene) appear to have reduced fertility in male “hybrids,” the resulting females were likely fully fertile (Sankararaman et al., 2014), permitting the transmission of Neanderthal, Denisovan and other alleles to the newcomers (Varki, 2016). These alleles influence numerous clinical traits in modern humans and have been implicated in both unfavourable medical conditions (immunological, dermatological, neurological, psychiatric and reproductive diseases) and advantageous modern human adaptations (enhanced sense of smell, high‐altitude adaptions, increased immune response and tougher skin and hair) (Vernot & Akey, 2014). Accordingly, and as genetic variations are important in the design, analysis and interpretation of epidemiological studies (Tishkoff & Kidd, 2004), assuming a more Afrocentric evolutionary perspective should contribute towards elucidating the coevolutionary relationships between hominins and pathogens in Africa.

Arriving in Eurasia years before BMHs, *Homo erectus* diverged genetically and phenotypically from our last common African ancestor. As groups of BMHs emerged from Africa after *c*. 65 ka, they overlapped spatially and temporally with these divergent groups. Whereas these encounters provided the impetus for genome admixture and the introgression of Neanderthal and Denisovan DNA into the genomes of newly arriving (African) BMHs (Racimo, Sankararaman, Nielsen, & Huerta‐Sánchez, 2015; Varki, 2016), the sexual transmission pathway also facilitated the transference of oncogenic viral and bacterial pathogens such as KSHV, HPV and *H. pylori* between these closely related groups. *Homo sapiens* departed Africa infected with, amongst other pathogens, *H. pylori* (Dimitriadi, 2014; Eusebi, Zagari, & Bazzoli, 2014) and KSHV (HHV‐8; Mancuso et al., 2013; Minhas & Wood, 2014). The former is a conspecific human pathogen which coevolved with ancestral southern African San hunter‐gatherers representing one of the deepest branches of the human population tree (Moodley et al., 2012). It is still implicated in the oncogenesis of >75% of gastric carcinomas (Plummer et al., 2016) and variant lineages are still evolving and spreading within Africa (Nell et al., 2013). KSHV is cited as a primary causative agent in ~100% of documented Kaposi's sarcoma, a cancer that develops from the cells that line lymph or blood vessels (Plummer et al., 2016). Both KSHV and *H. pylori* have featured prominently in pathogenic extinction theories concerning Neanderthals (Houldcroft & Underdown, 2016; Wolff & Greenwood, 2010).

Newly arriving African BMHs would have presented a formiddable reservoir of tropical diseases for the Neanderthal population of Eurasia. Because Neanderthals were adapted to a specific geographic infectious disease environment, exposure to new pathogens from Africa may have been catastrophic (Houldcroft & Underdown, 2016). As pathogens, including viruses, are prominent drivers of immune response adaptation in various mammalian species (Enard, Cai, Gwennap, & Petrov, 2016), genetic variation transmitted through admixture with Neanderthals represents a source of potentially advantageous variants (Quach et al., 2016). Neanderthals had, however, also conveyed novel strains of HPV (including the highly oncogenic HPV16) to BMHs (Pimenoff et al., 2016). The analyses of human and virus genomic data suggest that although HPV16 coevolved with African BMHs as a host population, HPV16 already infected the ancestral human populations more than 500 ka (Pimenoff et al., 2016). Accordingly, two main HPV16 lineages (A and BCD) codiverged with the Neanderthal and BMH populations. Following diversification of HPV16 variants amongst Neanderthal and Denisovan populations in Eurasia, novel HPV16A strains re‐infected newly arriving BMHs. HPV16 has since come to represent the most pervasive STI in the world (Bruni et al., 2010). Besides being implicated in ~100% of cervical cancer cases, it contributes to the incidence of other cancers affecting females and males, including anal, oral and pharyngeal and genital cancers (World Health Organization, 2010). The significance of this prehistoric HPV infection event is illustrated by the fact that cervical cancer still causes ~280,000 human deaths per year, with >80% of these occurring in countries with limited medical resources (Sankaranarayanan, Nessa, Esmy, & Dangou, 2012).

As in oncogenesis, viruses and bacteria are implicated in the majority of known historical epidemics. Since the first known influenza pandemic was described by Hippocrates in 412 BC (Singh & Misra, 2012), the world has experienced no less than 120 major disease epidemics. The Black Death, caused by the transmission of the *Yersinia pestis* bacterium to humans by fleas, resulted in the deaths of an estimated 75–200 million people in Europe from 1346 to 1353 (Bos et al., 2011). Nearly 400 years later, the Spanish influenza (caused by the H1N1 influenza A virus) pandemic of 1918–1919 resulted in the death of up to 100 million people across Europe, Asia and the Americas (Johnson & Mueller, 2002). During the 20th and 21st centuries, an additional 78 epidemic outbreaks occurred, 10 of which spread across the globe. In no more than 40 years, HIV/AIDS has killed 25 million people worldwide and malaria continues to kill ~2 million people per annum. Highly infectious diseases continue to plague the planet in both urban and rural locations. And, thus far, only two epidemic diseases, namely smallpox (*Variola major*) and rinderpest (*Morbillivirus*), have been eradicated by vaccines. Many others, including polio (*Picornaviridae*) and influenza (*Orthomyxoviridae*) have not yet been eliminated (Boire, Riedel, Parish, & Riedel, 2014; Klepac, Funk, Hollingsworth, Metcalf, & Hampson, 2015). In 2016, the WHO documented >120 novel re‐emerging disease outbreaks (http://www.who.int/csr/don/archive/year/2016/en/) including two novel (Sudanese haemorrhagic fever and American and Polynesian Guillain–Barré syndrome) epidemic pathogens (Table 2).

**Table 2:** Epidemic and pandemic zoonotic disease outbreaks as recorded by the WHO for 2016 which experienced 120 disease outbreaks involving 20 diseases comprising 228,612 reported cases and 13,026 human deaths (http://www.who.int/csr/don/archive/year/2016/en/)

	Disease (agent, transmission, reservoir)	Location(s)	Cases	Deaths	Fatality (%)
Epidemics	Influenza (H7N9 virus; domestic poultry, wild birds)	China	117	34	29
	Influenza (H5N6 virus; migratory waterfowl, domestic poultry)	China	10	0	0
	Monkeypox (*Orthopoxvirus*, monkeys, Gambian giant rats, squirrels)	Central African Republic	1	1	100
	Oropouche virus (*Orthobunyavirus*, sloths, midge‐borne, mosquito‐borne)	Peru	57	0	0
	Haemorrhagic fever (undiagnosed)	South Sudan	51	10	20
	*Elizabethkingia anophelis* (*Flavobacteriaceae*, environmental bacterial pathogen)	United States of America	57	0	0
	*Escherichia coli* O157:H7 (*Enterobacteriaceae*, environmental, food, intestinal)	United Kingdom	105	0	0
	Salmonellosis (*Enterobacteriaceae,* contaminated water, meat, poultry, eggs)	United States of America	124	0	0
	Cholera (*Vibrio cholera,* humans, environmental, seafood)	Tanzania	24,108	378	2
Pandemics	Plague (*Yersinia pestis* bacterium, vector [flea]‐borne)	United States of America	1	0	0
		Russia	1	0	0
		Madagascar	14	10	71
	Dengue fever (dengue virus [DENV], mosquito‐borne)	Burkina Faso	1,061	15	1
		Uruguay	20	0	0
	MERS‐CoV (*Coronavirus,* bats, dromedary camels)	Saudi Arabia	191	31	16
		Qatar	3	0	0
		Oman	1	0	0
		United Arab Emirates	3	3	100
		Bahrain	1	0	0
		Austria	1	0	0
		Thailand	2	0	0
	Polio (*Picornaviridae*, humans, rhesus monkeys, cynomolgus monkeys, African green monkeys)	Nigeria	3	0	0
		Laos	5	0	0
	Rift Valley fever (*Phlebovirus,* cattle, buffalo, sheep, goats, camels)	Niger	64	23	36
		China	1	0	0
	Chikungunya (*Alphavirus,* birds, rodents, mosquito‐borne)	Kenya	10	0	0
		United States of America	1	0	0
		Argentina	54	0	0
	Ebola viral disease (humans, African fruit bats)	Sierra Leone	14,124	3,956	28
		Liberia	10,675	4,809	45
		Guinea	3,811	2,543	67
		Mali	8	6	75
		Nigeria	20	8	40
		Senegal	1	0	0
		United States of America	4	1	25
		Spain	1	0	0
		Italy	1	0	0
		United Kingdom	1	0	0
Yellow fever (*Flavivirus,* mosquito‐borne)	Angola	3,850	797	21
	Democratic Republic of the Congo	1,304	129	10
	Uganda	30	7	23
	Kenya	2	1	50
	China	20	0	0
Lassa fever (*Arenavirus*, multimammate rats, other rodents)	Benin	54	28	52
	Nigeria	432	231	53
	Liberia	38	0	0
	Germany	3	0	0
	Togo	2	0	0
	Sweden	1	0	0
	Benin	71	2	3
Zika virus (*Flaviviridae*, mosquito‐borne, humans, primates)	Papua New Guinea	6	0	0
	Peru	1	0	0
	Saint Lucia	2	0	0
	Chile	1	0	0
	United States of America	2	0	0
	Brazil	165,907	0	0
	Vietnam	2	0	0
	Cuba	1	0	0
	France	1	0	0
	Argentina	1	0	0
	Sint Maarten	3	0	0
	Trinidad and Tobago	1	0	0
	Saint Vincent and the Grenadines	1	0	0
	Guadeloupe	1	0	0
	Bonaire	1	0	0
	Aruba	1	0	0
	United States Virgin Islands	1	0	0
	Dominican Republic	10	0	0
	Maldives	1	0	0
	Haiti	2	0	0
	Guyana	1	0	0
	Barbados	1	0	0
	French Guiana	2	0	0
	Ecuador	1	0	0
	Puerto Rico	1	0	0
	Bolivia	1	0	0	
Guillain–Barré syndrome (undiagnosed)	Panama	1	0	0	
	United States of America	2	1	50	
	French Polynesia	42	0	0	
	Columbia	86	0	0	
	Venezuela	252	0	0	
	Brazil	1,708	0	0	
	Martinique	2	0	0	
	El Salvador	46	2	4
Total			228,612	13,026	**–**

Current assessments of global evidence for prehistoric disease vectors and pathogens are providing increasingly informed perspectives on the range of pathogenic species and their geographic origins, and the phylogenetic relationships of extant pathogens suggest that many infectious diseases have been coevolving with humans for millennia (Houldcroft & Underdown, 2016). However, as a result of the fact that an integrated “One Health” approach, emphasizing the interconnectedness of human, animal and environmental health (Degeling et al., 2015; Gibbs, 2014), has not been applied to prehistoric human populations, current disease prevalence models provide inadequate information concerning the diseases that infected our sub‐Saharan African ancestors. Given the long evolutionary association between humans and pathogens in sub‐Saharan Africa, the systematic examination of disease organisms derived from prehistoric African contexts is essential.

## 5 THE ROLE OF SOUTHERN AFRICA IN PALAEOPATHOGENIC RESEARCH

The application of state‐of‐the‐art molecular analytical techniques to archaeological remains has transformed hominin evolutionary research. Examples of developments in the field of aDNA includes the recovery (from permafrost conditions) of aDNA from equid remains dated to ~700 ka (Orlando et al., 2013), the sequencing of the oldest human nuclear DNA (nDNA) from Sima de los Huesos (Spain) dated to 430 ka (Meyer et al., 2016) and the oldest‐known *H. sapiens* genome which was extracted from a human femur recovered from the banks of the Irtysh River in Siberia, dated to 45 ka (Fu et al., 2014). Molecular analytical techniques have also been applied to the emerging field of apDNA and have contributed significantly to understandings of historical epidemiological aetiology (Bos et al., 2015; Devault et al., 2014; Harkins & Stone, 2015; Rasmussen et al., 2015; Schuenemann et al., 2013). As an example, and given the ambiguity regarding the assignation of, for instance, *M. tuberculosis* or *Brucella melitensis* as causative agents of macromorphological skeletal features, the biomolecular (DNA) analysis of archaeological human remains has gained increasing recognition (Kay et al., 2014). Biomolecular techniques are not limited to the extraction of aDNA from skeletal remains (Meyer et al., 2016) and have also been applied to the analyses of archaeological sediments (Haouchar et al., 2014), human and animal coprolites (Cano et al., 2014) and curated museum specimens (Yeates & Gillings, 2016).

Southern Africa is perfectly positioned to play an essential role in current palaeopathogenic research. The region boasts an unrivalled techno‐cultural archaeological record spanning >2 million years and comprising >250 excavated and securely dated Late Pleistocene (125–12 ka) and Holocene (<12 ka) archaeological assemblages (Lombard et al., 2012). It is also here that, more than 32 years ago, the field of aDNA was launched with the publication of mitochondrial DNA (mtDNA) sequences derived from an extinct quagga (*Equus quagga*; Higuchi, Bowman, Freiberger, Ryder, & Wilson, 1984). This was followed, in 1985, by a report of the detection of human DNA in an extract of muscle from a pre‐Dynastic (2,430 years) Egyptian mummy (Pääbo, 1985), and, in 2005, the molecular characterization of the 1918 influenza virus by Taubenberger et al. (2005) which initiated the age of ancient pathogen genomics. Since then, the formerly nascent field of aDNA research has significantly altered our understanding of the human evolutionary story.

The likelihood of detecting ancient diseases, particularly from subtropical African conditions, poses some complications. Most diseases are entirely invisible in the archaeological record as they leave no indications of their presence on human skeletal remains (Houldcroft, Ramond, Rifkin, & Underdown, 2017). Even those that do affect skeletal morphology (e.g., *Y. pestis*,* M. tuberculosis*,* Mycobacterium leprae*,* T. pallidum*,* B. melitensis*,* P. falciparum*,* Trypanosoma cruzi*) are often misdiagnosed. Taphonomic alterations also mimic disease conditions which can induce interpretation errors (pseudopathologies), even for experienced palaeopathologists (Dutour, 2008). Accordingly, and unless detected with innovative archaeometric techniques such as X‐ray synchrotron microtomography (Odes et al., 2016; Randolph‐Quinney et al., 2016) or molecular (DNA) analyses, evidence symptomatic of ancient disease incidence is essentially imperceptible. Microorganisms also differ in the propensity of their DNA to decay and undergo physicochemical changes over time. nDNA degrades roughly twice as fast as mtDNA (Allentoft et al.,^2012^). Mycobacteria have highly resistant hydrophobic cell walls and DNA rich in guanine and cytosine. This confers greater molecular stability and allows these bacteria to physically persist for at least 250 years (Donoghue & Spigelman, 2006). Similarly, gram‐negative bacteria such as *Y. pestis* are characterized by cell envelopes comprising a peptidoglycan cell wall between an inner and outer cell membrane (Rasmussen et al., 2015), rendering these bacteria structurally robust. Conversely, *T. pallidum*, the causative agent of syphilis, is a spirochaete which is prone to structural deterioration. It is consequently not surprising that *M. tuberculosis*,* M. leprae* and *Y. pestis* are the subjects of the majority of ancient microbial pathogen studies. While the DNA of most bacteria and fungi are likely to be detected, viral DNA is less likely to be preserved and therefore detected (Houldcroft et al., 2017). Unlike the double‐stranded DNA of bacteria, viral genetic information is encoded in a variety of structures, including double‐ or single‐stranded DNA or RNA genomes. Viral aDNA is more likely to be preserved than viral aRNA because DNA degrades more slowly than RNA, except when integrated in the host genome (Arbuckle et al., 2010). Ancient single‐stranded or RNA genome viruses in archaeological samples may occur when preservation conditions are exceptional, for example in caves with cool and constant temperatures (Meyer et al., 2014) or where soft tissue has been preserved (Maixner et al., 2016).

But what are the implications of information concerning prehistoric pathogens for modern disease prevention and treatment strategies? And how can novel data concerning ancient pathogens and epidemics provide tangible benefits to living populations? These are important questions as the societal relevance of academic research is becoming an increasingly contentious topic. Few African tertiary institutions have the luxury of allocating funding to either established or novel research projects unless they are pertinent to the improvement of current societal issues. Human health and longevity, and the impact of infectious and noncommunicable diseases on human well‐being arguably represent one of the most important current societal issues, particularly in Africa. Bioarchaeological research is an expensive enterprise and it does not always attract the same degree of funding such as that driven by the need for economic growth and medical breakthroughs. First, information derived from the recovery, high‐throughput sequencing (HTS) and bioinformatic analyses of apDNA can possibly be used to anchor pathogen mutation rates and reconstruct viral and bacterial evolutionary processes. Second, and more controversially, it might also prove highly valuable in the development of new vaccines and, possibly, play a role in the discovery of novel pathogens that might pose significant future disease threats to humanity.

The information derived from HTS and bioinformatic analyses of apDNA can be used to anchor pathogen mutation rates and reconstruct viral and bacterial evolutionary processes. Genetic mutations occur through various mechanisms, including single nucleotide mutations, insertions or deletions and chromosome rearrangements. As mutations play an important role in pathogen evolution and virulence, information derived from apDNA sequences have much epidemiological potential. Genetic mutations and the rates at which they transpire are however very difficult to determine as they occur over both the long and the short term (Didelot, Walker, Peto, Crook, & Wilson, 2016). Moreover, both recombination and mutation rates vary substantially amongst pathogens (Warinner et al., 2017). While these mechanisms are important drivers of microbial genetic diversity, they complicate efforts to define species and to trace the evolutionary history of microbial lineages. Some studies have nevertheless addressed the age of bacterial pathogens that infected ancient humans, and many of these have provided significant insights into pathogen evolution. Comparative genomics can reconstruct short‐term evolutionary histories of pathogen clades whose diversity converges towards a most recent common ancestor (MRCA) that existed decades, centuries or even millennia ago (Achtman, 2016; Der Sarkissian et al., 2015). For example, following calibration of the evolutionary divergence within *H. pylori* against ancient human migrations, the MRCA of *H. pylori* approximates that of anatomically modern humans. The genetic diversity of *H. pylori* also reflects other human demographic events, including the peopling of the Americas and Asia (Nell et al., 2013). While the MRCAs of bacterial pathogens such as *M. tuberculosis* and *Y. pestis* span some 6,000 years, comparative genomics of modern isolates suggests that these bacteria also spread across the globe following human dispersals from Africa during the Pleistocene. The characterization of historical *Y. pestis* strains and their comparison to extant strains provide insight into the role of bacterial evolution in epidemiological virulence and communicability (Rasmussen et al., 2015). The genome sequencing of *Y. pestis* indicates a MRCA at *c*. 55 ka. Although this pathogen was present in Europe and Asia as long as 5 ka, it only acquired the ability to use fleas as an effective mode of vectored transmission some 3 ka. This was the result of a single genetic mutation (YMT) affecting pathogen virulence and the ability of the bacterium to colonize and survive in flea intestines. While plague might have been less transmissible without arthropod vectors (fleas), it would still have been lethal as >90% of untreated cases of pneumonic plague is fatal. Prehistoric pathogen research can therefore contribute to our understanding of disease evolution by providing time‐stamped sequence data and by allowing the re‐evaluation of hypotheses regarding the extent of our coevolutionary history with pathogenic and commensal organisms (DeWitte, 2016).

Genomics has also enabled the use of entire pathogen genomes to search for protective antigens that were impossible to identify with conventional technologies. Following the successful development of a vaccine against smallpox (*V. major*) by Edward Jenner in 1796 (Funkhouser, 2010), the 20th and 21st centuries has witnessed several successes in vaccination campaigns against infectious diseases. Recent genomic data have been used to identify vaccine antigens for specific *Escherichia coli* strains (e.g., the K1 strain implicated in the onset of neonatal meningitis), *Neisseria meningitidis* (the causative agent of meningitis and other meningococcal diseases) and *T. pallidum* (the causative agent of syphilis, yaws, etc.). The vaccine ST‐246 was developed using pathogenic *Orthopoxvirus* (smallpox, cowpox, monkeypox, etc.) genomic data and used to treat a child who developed life‐threatening eczema vaccinatum (DeWitte, 2016; Grimm & Ackerman, 2013). As recently as 2015, the first vaccines against malaria (Mosquirix) (O'Hagan & Fox, 2015), Ebola (rVSV‐ZEBOV) (Henao‐Restrepo et al., 2015) and dengue fever (Dengvaxia) (Pang, 2016) have been approved for human trials and inoculation. The vaccine against Meningococcus B (licensed in Europe in 2013 and the USA in 2015) is a pertinent example of prototype vaccine developed by genome‐based approaches or “reverse vaccinology” (Giuliani et al., 2006).

Whether ancient pathogen genome data can be put to comparable use is not yet clear, largely because the influence of microbial physical characteristics and the susceptibility of DNA and RNA to postdepositional degradation is unpredictable (Maixner et al., 2016; Rasmussen et al., 2015). There is, however, a single known instance of the utility of “old” (not exactly ancient as such) pathogenic DNA data in the development of modern vaccines. The genome sequencing of the 1918–1919 Spanish influenza (H1N1) virus yielded novel insights into influenza biology and pathogenesis (Reperant, Kuiken, & Osterhaus, 2012; Taubenberger et al., 2012). H1N1 emerged in 1893 and, by 1918, the virus had accumulated ~375 mutations (i.e., ~15 mutations per year). One of these entailed the acquisition of mutations derived from the H5N1 avian virus, and the result was the 1918 Spanish influenza pandemic. The ensuing pandemic viruses of 1957, 1968, and 2009 all descended from the original 1918 virus. Significantly, genome sequencing of the 1918 virus provided the basis for understanding that the key 2009 virus HA gene, after being transmitted from humans to pigs in ~1918, was maintained in pigs for nearly 90 years as a separate lineage from the 1918 virus (Taubenberger et al., 2012). This knowledge contributed to ongoing research to develop universal influenza vaccines for all known and potentially emerging pandemic influenza viruses and facilitated the rapid assessment of the potential virulence of the 2009 H1N1 pandemic virus (Medina et al., 2010). Interestingly, 1918 Spanish influenza virus‐specific B cell clones could still be recovered from elderly survivors 90 years after their exposure to the virus but before their exposure to the 2009 pandemic virus (Taubenberger et al., 2012). These findings provided a scientific rationale for targeting the initial 2009 H1N1 pandemic vaccine to those who needed it most, namely predominantly younger persons who had not been exposed to the cross‐protective 1918 virus or to its seasonally prevalent descendants. Thus, early in the 2009 pandemic, limited vaccine supplies that might have been misdirected to the traditional risk group (the elderly) were instead administered to younger persons, who benefitted most.

## 6 CONCLUSION

It is evident that ancient biomolecular research can contribute to existing genome databases which may have public health benefits by providing tools for developing therapeutics, particularly if virulent forms of ancient diseases re‐emerge. This is important as history has taught us that disease is by far the most effective eradicator of our species. Past pandemics are much more than just ancient history. They are important drivers of human genetic diversity and natural selection (Pittman, Glover, Wang, & Kol, 2016). It is also clear that the long‐term tracing of genetic adaptations and rates of evolutionary change are highly informative in understanding how a pathogen becomes virulent or transmissible, providing insights into how we can effectively manage future epidemics (Andam, Worby, Chang, & Campana, 2016; Boire et al., 2014).

DNA preservation is widely cited as a primary limiting factor pertaining to aDNA from tropical and subtropical African contexts, and most studies are based on finds from Northern Hemisphere and predominantly permafrost contexts (Haile et al., 2009; Kistler, Ware, Smith, Collins, & Allaby, 2017). Temperate and Arctic regions have generally yielded more aDNA sequences than tropical regions, partly because conditions are more favourable to the preservation of aDNA, but also because they have been sampled more intensively (Slatkin & Racimo, 2016). However, the recovery of human nuclear aDNA from Sima de los Huesos (Meyer et al., 2016) and human mtDNA from Mota Cave in Ethiopia (at 4.5 ka) (Llorente et al., 2015, 2016) and St. Helena Cave, South Africa (at 2.3 ka) (Morris, Heinze, Chan, Smith, & Hayes, 2014) suggests that chronological age does not predict DNA fragmentation and that aDNA and apDNA preservation is not contingent exclusively on subzero temperatures (Kistler et al., 2017). The prospect of retrieving both human and apDNA from sub‐Saharan African contexts is increasingly promising.

The past provides a prologue for discussions regarding emerging diseases, whether it concerns the biological origins of a potential pandemic or its social repercussions (Heymann, 2007). Disease epidemics are not new and they will continue to affect and potentially devastate human populations. Significantly, the exclusive focus on diseases that have emerged within the past decades is cited as responsible for the lack the temporal depth necessary to examine the changes in the behaviour of emerging diseases and the long‐term interactions between pathogens and human hosts (DeWitte, 2016). The severe economic and social repercussions of disease epidemics are clearly demonstrated by historical (e.g., plague, smallpox and influenza) and current (i.e., Zika, Ebola and SARS) examples. But the biological origin of a many prehistoric, historical and even contemporary causative pathogens remains mysterious. The emphasis should therefore also be on the development of sub‐Saharan capabilities to detect, predict, prevent and control all potential infectious disease epidemics rather than waiting for known diseases to threaten global human health. This is particularly important given the current global interconnectedness, which can put people at risk of diseases that emerge in distant locales. In addition, the discovery and re‐animation of two 30,000‐year‐old viruses (*Pithovirus sibericum* and *Mollivirus sibericum*) from Siberian permafrost (Legendre et al., 2015) highlights the severity of the impact that an increasingly warmer globe might have on pathogen prevalence (Wu, Lu, et al., 2016). Warmer temperatures and increased rainfall readily facilitate the introduction of new species of plants, animals and also microorganisms, altering the composition and dominance patterns of existing communities and increasing the susceptibility of humans to re‐emerging and even novel pathogens (Pauchard et al., 2015). Current climate models consistently predict increasingly suitable climatic conditions for endemic malaria transmission in Central Europe and North America (Caminade et al., 2014), and even in Northern Europe, pathogenic bacteria such as *Vibrio cholerae* (Baker‐Austin, Trinanes, Gonzalez‐Escalona, & Martinez‐Urtaza, 2017) are becoming increasingly prevalent. In the Southern Hemisphere and in sub‐Saharan Africa in particular, there is a direct correlation between increasing rainfall, warmer temperatures and the prevalence of infectious and also vector‐borne diseases, including malaria, trypanosomiasis, schistosomiasis, chikungunya and plague (Rosenthal, Ostfeld, McGarvey, Luriea, & Smith, 2015; Stensgaard, Booth, Nikulin, & McCreesh, 2015). This realization corroborates the significance of information derived from palaeopathogenic research on sub‐Saharan African archaeological contexts.

Because of the paucity of aDNA sequences from Africa, these novel pathogen genomes will be highly valuable and decidedly revealing, providing novel revelations concerning human‐pathogen coevolutionary processes (Slatkin & Racimo, 2016). The unique combination of an unrivalled archaeological record and a thriving and highly skilled academic community therefore places southern African archaeologists, geneticists and medical scientists in a prime position to explore past pathogenic influences and to contribute to the improvement of human health and longevity.
